# Endemic Gastrointestinal Anthrax in 1960s Lebanon: Clinical Manifestations and Surgical Findings

**DOI:** 10.3201/eid0905.020537

**Published:** 2003-05

**Authors:** Zeina A Kanafani, Antoine Ghossain, Ala I Sharara, Joseph M. Hatem, Souha S Kanj

**Affiliations:** * American University of Beirut Medical Center, Beirut, Lebanon; † Saint-Joseph University, Beirut, Lebanon

**Keywords:** anthrax, intestinal, Lebanon, ascites, spore-forming organisms, synopsis

## Abstract

Anthrax is an ancient disease caused by the gram-positive *Bacillus anthracis*; recently, it has gained much attention because of its potential use in biologic warfare. Anthrax infection occurs in three forms: cutaneous, inhalational, and gastrointestinal. The last type results from ingestion of poorly cooked contaminated meat. Intestinal anthrax was widely known in Lebanon in the 1960s, when a series of >100 cases were observed in the Bekaa Valley. We describe some of these cases, introduce the concept of the surgical management of advanced intestinal anthrax, and describe some of the approaches for treatment.

Several reports and reviews have recently shed light on anthrax and its cutaneous and pulmonary manifestations, focusing on its threat as a biologic weapon. The third form of the disease, gastrointestinal anthrax, has not received as much attention, and the research describing its manifestations is scarce. We report on several cases of intestinal anthrax from 1960 to 1974 in the Bekaa Valley in Lebanon, where the consumption of raw or poorly cooked meat is customary. One of the authors (A. Ghossain) treated and operated on all the patients described here. We describe a series of gastrointestinal anthrax cases, the reporting of which has become paramount with the current renewed interest in this entity, the scarcity of information in the research on this particular form of anthrax, and the high fatality rate in advanced disease. The cases described in this report were chosen to illustrate the protean manifestations of gastrointestinal anthrax.

## Clinical Manifestations and Surgical Findings

In March 1960, an acute and particularly severe abdominal syndrome was recognized in the Bekaa Valley in Lebanon. The first cases were described in male patients from 4 to 25 years of age with an illness that consisted of three phases. Phase I was marked by fainting spells, asthenia, low-grade fever, and headache. Patients rarely sought medical treatment at this stage because the symptoms were not serious enough. In the few patients seen at this stage, physical examination disclosed flushing of the face and red conjunctivae, but appearance was not otherwise affected. The first impression of a general practitioner who saw patients during this phase of illness was that the patient had an early viral infection. Phase II started when, 24 hours later, early symptoms persisted, and abdominal pain of variable intensity supervened. The pain description ranged from mild to severe paroxysms to constant pain. Low-grade fever, nausea, and vomiting were frequent; diarrhea, if present, was mild. Physical examination usually showed a smooth, ill-defined mass in the right lower quadrant or the periumbilical area and abdominal distention. At that stage, patients were referred to a specialist for acute abdominal infection. Three clinical findings, however, did not fit this diagnosis: 1) the illness started with vague, generalized symptoms instead of abdominal pain; 2) evidence of ascites was found on examination, 3) and patients had severe weakness and intravascular depletion, findings uncommon in early appendicitis.

Phase III was recognized because most patients were referred at that stage with rapid increase in abdominal girth and paroxysms of abdominal pain. Occasionally, gastrointestinal bleeding concurred. Upon examination, some features were frequently present: shock, ascites, flushed face, and red conjunctivae. Because of unclear and questionable diagnosis, exploratory laparotomy was performed on several patients, invariably showing an abundant yellowish and thick ascitic fluid, soft hypertrophied mesenteric lymph nodes (3 cm–5 cm) mostly in the ileocecal region, and substantial edema involving one segment of small bowel, cecum, or ascending colon. When the diseased segment was not resected during surgery, many patients experienced a rapid but transient postoperative clinical improvement. Ascites, however, soon reaccumulated, and most of these patients died from an overwhelming state of shock. Definite and steady recovery was observed in most patients who underwent partial bowel resection. Examination of the bowel segment at surgery or autopsy disclosed a central necrotic lesion (2 cm–3 cm) encircled by small soft red nodules (0.5 cm–1 cm in diameter), and surrounded by severe edema of the bowel wall with areas of hemorrhage. Bowel perforation was uncommon, as patients usually died before that progression (*1*,*2*).

Most of the patient population had these signs and symptoms. In some cases, however, two unusual clinical pictures were observed: the surgical form and abortive form. In the surgical form, the illness started with severe abdominal pain without the generalized symptoms of phase I. Surgery was often performed with the provisional diagnosis of acute infection in the abdomen. In the abortive form, the illness was limited to phase I, probably because such patients had taken penicillin or other antibiotics, thus interrupting the cycle of the disease (*2*,*3*).

## Epidemiologic Investigation

More cases were being described, but no clear diagnosis could be made until October 1960, when the cause of this curious clinical syndrome was elucidated. A 30-year-old woman with abdominal symptoms and ascites underwent laparotomy but died 12 hours later. Surgery showed ascites, an edematous small bowel loop, and huge mesenteric lymph nodes. Intestinal resection and lymph node biopsies were performed. Pathologic examination showed a central mucosal necrotic lesion along with hemorrhage and extensive edema. Gram-positive bacilli in chains were also observed. Bacterial cultures from blood and excised lymph nodes grew *Bacillus anthracis*. Inoculation of test animals resulted in death within 20 hours; their spleens had large amounts of anthrax bacilli (*4*). The identity of the bacillus was further confirmed when tissue specimens were sent to the Centers for Disease Control and Prevention (CDC) for analysis.

With this new information at hand, investigations were conducted to pinpoint the source of infection. The patient’s husband, a shepherd, was questioned and admitted that 4 days before the onset of his wife’s illness, he had slaughtered a dying goat. The meat was consumed raw by friends and family members (customary in some remote villages). All persons who ate meat were identified. The patient’s sister-in-law had had cutaneous anthrax above the lip and had an uneventful course with penicillin therapy. Another person had complained of vague abdominal pain and mild diarrhea; stool culture was negative. The goat’s skin was examined and found to have anthrax spores. Medical records of previous patients were then reviewed; their families were contacted, and a number of persons did recall eating raw meat few days before symptoms began. Furthermore, paraffin-embedded tissue blocs from a patient who died earlier of a similar illness were reexamined; evidence of gram-positive bacilli was found in the intestinal tissue (*4*).

Over 100 cases of intestinal anthrax were subsequently observed during a period of 14 years. Most patients were shepherds or their relatives who lived in remote villages of the Bekaa Valley. Their livestock was not vaccinated against anthrax, and they regularly lost some of their herds to a sickness they called “the disease of the spleen” because that organ was always massively swollen. Sick animals were slaughtered; the skin was sold, and meat was often ingested raw or inadequately cooked. The liver was usually eaten by children and young adults.

## Clinical Cases

### Case 1

In August 1961, a 26-year-old man complained of abdominal pain, nausea, vomiting, and generalized weakness of 4 days’ duration. Upon admission to the hospital, he was in a state of shock but was afebrile. He had a history of ingestion of raw meat 10 days before the symptoms began. On physical examination, he had abdominal tenderness and evidence of ascites. Analysis of the ascitic fluid showed an albumin level of 6.0 g/L and gram-positive rods with central spores. Cultures from the fluid grew *B. anthracis*. Attempts at resuscitation with intravenous fluids and treatment with penicillin failed, and the patient died 21 hours after admission.

### Case 2

A 24-year-old shepherd was admitted to the hospital in September 1961 with severe nausea, vomiting, and abdominal pain. His illness had started 1 week earlier with headache and mild abdominal pain. He reported eating raw meat from an ill goat. His younger brother, who ate from the same goat meat, had died of intestinal anthrax on the same day of admission as this patient. His pulse was 120 bpm, systolic blood pressure 95 mm Hg, and temperature 37.5°C. The patient had erythema and edema of the face, and a healing eschar on the lower lip that had reportedly started as a small vesicle. He had diffuse abdominal tenderness. His leukocyte count was 12,500 cells/mm^3^, with 73% neutrophils. Blood cultures grew *B. anthracis*. The patient received intravenous penicillin and improved. He was discharged 1 week later in good condition.

### Case 3

A 7-year-old boy was admitted in July 1965 with periumbilical pain, vomiting, and low-grade fever. He had eaten raw meat from an ill goat. His abdomen was distended, with a palpable mass in the right iliac fossa and evidence of ascites. His systolic blood pressure was 80 mm Hg. His leukocyte count was 13,000 cells/mm^3^, with 86% neutrophils. The presumptive diagnosis was acute appendicitis. He underwent laparotomy, which yielded approximately 2 L of fluid. The cecum and ascending colon were edematous, with a hemorrhagic mucosa; mesenteric lymphadenopathy was noted. Two days earlier, his mother reported a skin lesion over the left eyelid and severe surrounding edema suggestive of cutaneous anthrax. The patient was treated with penicillin but had persistent high-grade fever until day 9 of admission, when he was no longer febrile and was then discharged in good condition. In this particular case, the clinical and surgical findings suggested intestinal anthrax, although no microbiologic evidence was obtained.

### Case 4

**Figure 1:**
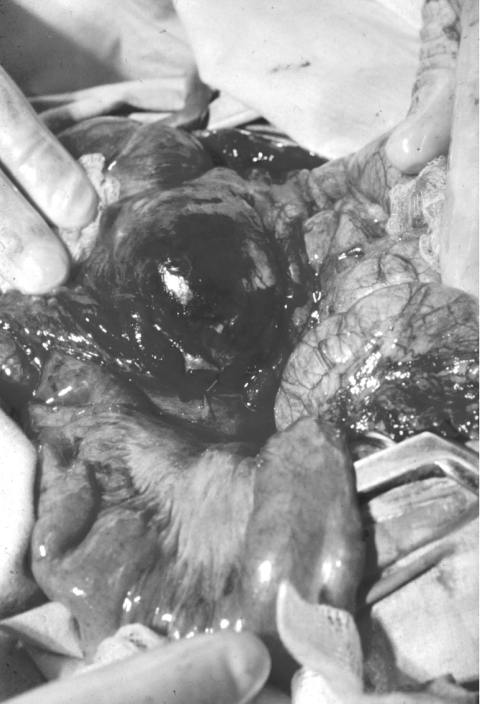
Extensive edema and hemorrhage involving the cecum in a patient with intestinal anthrax.

**Figure 2:**
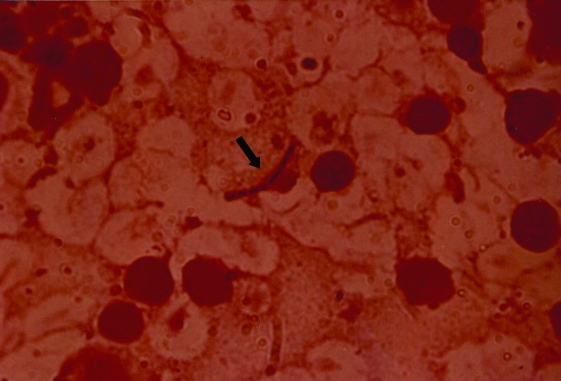
Anthrax bacilli (arrow) within mesenteric lymph node tissue.

A 17-year-old man was admitted to the hospital in October 1962 with abdominal pain, generalized weakness, and high-grade fever; he had eaten raw meat. On examination, the abdomen was distended with ascites, with tenderness in the right lower quadrant. The patient underwent laparotomy for suspected acute appendicitis that showed edema of the cecum and ascending colon, as well as enlargement of the mesenteric lymph nodes (Figure 1). Lymph node tissue, as well as blood and ascitic fluid cultures, grew *B. anthracis*. Gram-positive rods were visible upon pathologic examination of the excised lymph nodes (Figure 2). After surgery, the patient received penicillin and streptomycin. He improved slowly and was discharged on day 18.

### Case 5

In March 1974, a 15-year-old boy arrived at the hospital in shock. He had ascites and oculofacial congestion. Five days earlier, he had consumed raw meat from a sick goat. Despite aggressive resuscitation and treatment with penicillin, he remained in critical condition. Exploratory laparotomy revealed extensive edema of the cecum with mesenteric lymphadenopathy. Right hemicolectomy with primary anastomosis was performed along with continuous closed peritoneal drainage. His condition improved dramatically, and he was discharged 10 days later. Cultures from the mesenteric lymph nodes grew *B. anthracis*.

### Case 6

**Figure 3:**
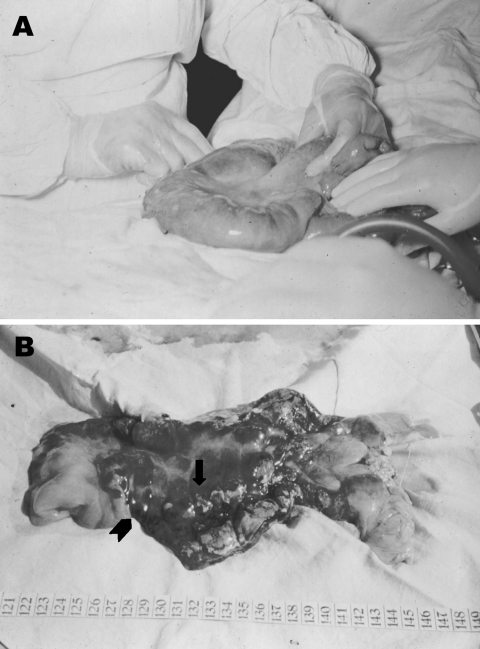
A. Severe edema of a small bowel loop in intestinal anthrax with a large mesenteric lymph node held between the surgeon’s fingers. B. Same segment of bowel opened after resection. Edema, necrosis, and mucosal hemorrhages exist. A central eschar (arrow) and small surrounding nodules (arrowhead) are reminiscent of the cutaneous lesions of anthrax.

A 20-year-old woman was admitted to the hospital in September 1974 with a 2-day history of abdominal pain after eating poorly cooked meat from a dying goat a week earlier. She was in a state of shock and had abdominal distention and a doughy mass in the periumbilical area. Laparotomy showed a large amount of ascitic fluid, an edematous small bowel loop proximal to the cecum, and enlarged mesenteric lymph nodes (Figure 3A and B). Intestinal resection and continuous drainage of the ascites were performed. Cultures from intraoperative lymph node samples grew *B. anthracis*. The patient improved gradually and was discharged 12 days later.

## Discussion

Gastrointestinal anthrax is extremely rare in the United States and western Europe but is more frequently encountered in developing countries around the globe. Research describing the clinical manifestations of this entity is scarce, poorly detailed, and inaccurate about appropriate management (*5*).

We performed a MEDLINE search to evaluate data published on gastrointestinal anthrax. Research that could be accessed and reviewed consisted of 11 reports from 1970 to 2000, most of which described single cases. Most patients were from developing countries, namely Iran, Bangladesh, Zimbabwe, Thailand, Uganda, India, and Turkey (*6*–*15*) (Table). Only two cases were reported from the United States, by CDC in 2000 (*16*). The infection was uniformly associated with eating contaminated meat, although theoretically, any ingested item could act as a vehicle for the transmission of anthrax spores. One study by Ndyabahinduka et al. was of an epidemic of gastrointestinal anthrax in Uganda in 1984, which affected 143 of 155 persons who ate meat from an infected zebu (Asian ox). In most cases, symptoms were those of gastroenteritis, with abdominal tenderness, vomiting, and diarrhea. Three children had blood-tinged stools from which anthrax bacilli were isolated. Thirteen patients had pharyngeal edema of variable severity. A fatal outcome from fulminant gastroenteritis was reported in nine patients, all children. All other patients responded quickly to tetracycline or penicillin (*10*). Another study by Phonboon et al. described an outbreak of gastrointestinal anthrax after an outbreak in cattle in Thailand; 74 persons become ill, and 3 died (*9*).

**Table:** Reports of gastrointestinal anthrax published from 1970 to 2000

Y	Authors (Reference)	Country	No. of patients	Disease location	Treatment	Outcome	
1970	Dutz et al. (7)	Iran	1	Stomach	Antibiotics	Died	
1977	Nalin et al. (8)	Bangladesh	1	Unspecified	Antibiotics	Survived	
1980	Jena (9)	Zimbabwe	1	Ascending colon	Surgery	Survived	
1984	Phonboon et al*.* (10)	Thailand	74	Unspecified	Unspecified	3 died	
1984	Ndyabahinduka et al*.* (11)	Uganda	143	Unspecified	Antibiotics	9 died	
1985	Bhat et al*.* (12)	India	1	Unspecified	Antibiotics	Died	
1990	Sekhar et al. (13)	India	20 internal*	Unspecified	Antibiotics	Unspecified	
1990	Kunanusont et al. (14)	Thailand	3	Stomach	Antibiotics	1 died	
1995	Alizad et al. (15)	Iran	1	Unspecified	Antibiotics	Died	
1997	Tekin et al. (16)	Turkey	1	Cecum and ascending colon	Surgery	Survived	
2000	CDC (17)	USA	2	Unspecified	Antibiotics	Survived	

^a^Internal includes inhalational anthrax, gastrointestinal anthrax, anthrax meningitis and septicemia; CDC, Centers for Disease Control and Prevention.

In published cases of gastrointestinal anthrax, death was more common in patients who had severe symptoms, including hematemesis, vomiting, abdominal pain, and distention (phase III), and who were only treated with antibiotics (*6*,*11*,*13*,*14*). Surgical exploration and bowel resection was performed in two patients first seen in phase III (*8*,*15*). The disease involved the ascending colon alone in one case and the cecum and ascending colon in the other. After surgery and antibiotic therapy, both patients recovered and were discharged. These two cases illustrate the benefit of surgery in the advanced form of gastrointestinal anthrax. These findings support our current approach for managing patients whose condition remains unstable after 6 to 12 hours of treatment, namely, administering antibiotics and adequate resuscitation and then resecting the diseased bowel segment. The rationale behind surgical resection is to overcome not only the large bacterial load but also the larger load of toxin in diseased tissues. In all our cases, the disease was confined to a single bowel segment, mostly small bowel or cecum. The clinical condition of patients improved rapidly and steadily after resection. Initial results were disappointing, since several patients died postoperatively of anastomotic leaks, dehiscence, and fistulization. Incomplete resection, severe hypoproteinemia, and rapid reaccumulation of protein-rich ascitic fluid were the main causes of surgical failure.

Based on our experience, the approach used in the management of cases of gastrointestinal anthrax should consist of: 1) initiation of intensive intravenous antibiotic therapy as soon as the diagnosis is made, 2) wide resection into seemingly healthy tissues with primary anastomosis in patients who did not improve with medical therapy, 3) continuous drainage of the ascites, as fluid will continue to accumulate for several days after surgery, 4) and aggressive replacement of protein and electrolyte losses (*2*,*17*). However, to make any generalization about the preferred mode of treatment of gastrointestinal anthrax in the absence of solid and reproducible clinical and epidemiologic data would be difficult. Furthermore, with the current improved access to medical care and advances in diagnostic techniques and supportive measures compared to the 1960s, surgical intervention might now be limited to few cases of advanced disease unresponsive to medical therapy.

Following the 1960s outbreak, some areas were recognized as being contaminated by anthrax spores. Grazing of livestock in these “damned fields” (as they were called by ancient French farmers) has since been avoided by shepherds, thereby virtually eliminating the disease from the Bekaa Valley (*2*,*3*).

## Conclusion

We describe the clinical spectrum of gastrointestinal anthrax, a disease that was endemic in Lebanon in the 1960s. We also report on the success of surgical treatment in some of the advanced cases, emphasizing the vital role of aggressive supportive measures in patient management. Our report lacks detailed epidemiologic data describing the incidence, age and sex distribution, and outcome of patients because most cases date back to the 1960s, a time when epidemiologic studies were scarce in a developing country such as Lebanon. Our experience with gastrointestinal anthrax, however, remains valuable because of the rarity of this condition and the dearth of data on management approaches.

Naturally, the consumption of raw meat, still widely practiced in many countries, should be strongly discouraged through education of the mass population as to the health hazards associated with such a custom. Ultimately, however, the control of anthrax in humans is chiefly dependent on control of the disease in animals. In the event of an anthrax enzootic, extensive investigations should be conducted to identify the source of infection and eliminate it. Milk from areas experiencing enzootics should be discarded, and sick animals should be isolated. Affected farms should be quarantined for at least 2 weeks after the last death from anthrax. The release of *B. anthracis* in the environment should be avoided to prevent future disease; dead animals should be burned or buried deeply and covered with lime. Their carcasses should not be slaughtered or necropsied, as exposure of the vegetative forms to the ambient atmosphere enhances sporulation. Annual vaccination of livestock and avoidance of clandestine slaughtering remain vital to the prevention of disease in endemic areas (*18*–*20*).
